# The Relationship between Influenza Vaccination Habits and Location of Vaccination

**DOI:** 10.1371/journal.pone.0114863

**Published:** 2014-12-09

**Authors:** Lori Uscher-Pines, Andrew Mulcahy, Jurgen Maurer, Katherine Harris

**Affiliations:** 1 RAND Corporation, Arlington, Virginia, United States of America; 2 University of Lausanne, Lausanne, Switzerland; 3 IMPAQ International, Washington, D.C., United States of America; Melbourne School of Population Health, Australia

## Abstract

Although use of non-medical settings for vaccination such as retail pharmacies has grown in recent years, little is known about how various settings are used by individuals with different vaccination habits. We aimed to assess the relationship between repeated, annual influenza vaccination and location of vaccination. Study Design: We conducted a cross-sectional survey of 4,040 adults in 2010. Methods: We fielded a nationally representative survey using an online research panel operated by Knowledge Networks. The completion rate among sampled panelists was 73%. Results: 39% of adults reported that they have never received a seasonal influenza vaccination. Compared to those who were usually or always vaccinated from year to year, those who sometimes or rarely received influenza vaccinations were significantly more likely to be vaccinated in a medical setting in 2009–2010. Conclusions: Results indicate that while medical settings are the dominant location for vaccination overall, they play an especially critical role in serving adults who do not regularly receive vaccinations. By exploring vaccination habits, we can more appropriately choose among interventions designed to encourage the initiation vs. maintenance of desired behaviors.

## Introduction

Annual vaccination is the most effective way to prevent the transmission of influenza. Yet, in recent years only about 40% of adults in the U.S. were vaccinated annually [1]. A key challenge facing the public health community is to identify and implement effective and efficient strategies for improving influenza vaccination rates. One prominent approach is to increase access to vaccinations at non-medical, complementary settings such as retail settings and workplaces [2], [3].

While we have extensive data on the various locations where individuals seek vaccination and the importance of physicians’ offices as a source of counseling on vaccination [4]–[7], we have little information about how various settings are used by individuals with different vaccination habits (e.g., occasional vaccination vs. repeated, annual vaccination) and how preferred locations change as individuals become more accustomed to regular vaccination.

To address this gap, we used a large, nationally representative sample of U.S. adults to study the association between vaccination habits, based on self-reported experience with influenza vaccination, and vaccination location. Our investigation provides descriptive information about the potential roles that complementary settings play in promoting the initiation of vaccination among those who have never been vaccinated and in promoting the maintenance of previously established vaccination habits.

## Materials and Methods

We fielded a nationally representative survey of U.S. adults age 18 and older (n = 4,040) between March 5–24, 2010 using an online research panel operated by Knowledge Networks (KN). KN recruits panelists through a probability-based sampling method that include both online and offline households [8], [9]. To ensure diversity, we oversampled older panelists, Blacks, and Hispanics (Table 1). The completion rate among sampled panelists was 73%.

**Table 1:** Overview of selected sample characteristics (n = 4,040)*.

Characteristic	Unweighted n	Weighted % (95%-CI)
	(reflecting stratification)	(using poststratification weights*)
**Age**		
18–29	297	21.7 (19.0;24.3)
30–44	525	28.2 (25.4;31.1)
45–59	1,359	25.6 (23.4;27.8)
60+	1,859	24.5 (22.6;26.3)
**Race/Ethnicity**		
White	1,808	68.7 (66.2;71.2)
Black	1,141	11.3 (9.9;12.7)
Hispanic	588	13.5 (11.5;15.6)
Other/Mixed	503	6.5 (5.2;7.8)
**Sex**		
Male	1,880	48.3 (45.5;51.1)
Female	2,160	51.7 (48.9;54.5)
**Education**		
Less than high school	374	13.4 (11.4;15.4)
High school	1,032	30.7 (28.1;33.3)
Some college	1,347	28.4 (25.9;30.9)
College degree or higher	1,287	27.5 (25.1;29.9)
**Work status**		
Employed	1,570	48.6 (45.8;51.4)
Self-employed	288	6.5 (5.2;7.9)
Temporary leave	46	2.0 (0.9;3.1)
Unemployed	245	8.5 (6.9;10.1)
Retired	1,257	16.4 (15.0;17.9)
Disabled	422	10.1 (8.5;11.7)
Other non-working	212	7.8 (6.3;9.4)
**Vaccination Frequency**		
Never	1,248	39.1 (36.3;42.0)
Rarely**	461	11.2 (9.5;12.9)
Sometimes**	126	3.3 (2.3;4.4)
Always/Usually**	1587	31.4 (28.9;33.8)
First time in 2009–2010	136	3.9 (2.8;5.0)
First time in 2008–2009	258	5.6 (4.4; 6.8)
First time in 2007–2008	193	5.2 (3.8–6.6)
**Internet access**		
(prior to panel recruitment)		
Yes	1,097	64.5 (61.7;67.3)
No	2,943	35.5 (32.7;38.3)

*The poststratification weights were computed using data from the Current Population Survey and adjust for known sampling probabilities; sample stratification; and non-response to panel recruitment and panel attrition.

**Calculated for only those respondents who reported receiving their first influenza vaccination three or more years prior to the fielding of the survey.

In addition to eliciting influenza vaccination status for the 2009–2010 season for both influenza vaccinations available at the time (H1N1 and seasonal), we asked respondents a series of questions regarding their previous receipt of seasonal influenza vaccinations as follows: “Not counting this past flu season (August 2009–March 2010) have you ever been vaccinated against seasonal flu in the past?”, “When was the first time you got a seasonal flu vaccine?” and “How often do you get a seasonal flu vaccine (every year, most years, some years, rarely)?” Based on responses to these questions, we classified respondents into three categories: (1) never vaccinated; (2) vaccinated for the first time during the last three years prior to the survey; (3) vaccinated for the first time more than three years prior to the survey. We defined those in the third category as having a “vaccination history” and further classified them as always or usually, sometimes, or rarely vaccinated.

We assessed whether respondents were recommended for seasonal vaccination using self-reported information on age, having one or more high-risk health conditions or being pregnant, occupational status (e.g., being a healthcare worker), and having young children or household contact with high-risk individuals [10].

Pearson’s chi squared tests were used to calculate p-values. We also used logistic regression to assess the association between vaccination history and vaccination in a medical setting adjusting for age, gender, income, race, and membership in a group specifically recommended for seasonal influenza vaccination. Because the substantive interpretation and statistical significance of the associations remained unchanged after adjustment, we report unadjusted analyses for ease of interpretation.

All analyses were conducted using Stata 11 (StataCorp, College Station, TX). Post-stratification weights were used to adjust for known sampling probabilities, sample stratification, non-response to panel recruitment, and panel attrition as well as to ensure that our final data matched selected socio-demographic characteristics of the U.S. adult population including gender, age, race/ethnicity, education, region, metropolitan area, internet access, and language spoken at home [11]. Our study was approved by RAND’s Institutional Review Board.

## Results

Thirty-nine percent of adults reported that they have never received a seasonal influenza vaccination (Table 1). Among those who reported never having received a seasonal influenza vaccination, 64% were specifically recommended for annual vaccination based on 2009–2010 Advisory Committee of Immunization Practices (ACIP) recommendations in place at the time. Based on multiple survey questions about vaccination history described above, we calculated that in each of the flu seasons between 2007–2010, approximately 4–6% of U.S. adults were vaccinated for the first time. Furthermore, among adults with a history of influenza vaccination, 68.4% reported receiving a vaccination every/most years, 7.3% reported receiving a vaccination some years, and 24.4% reported rarely receiving an influenza vaccination.

39% of adults were vaccinated for seasonal influenza in 2009–2010. Among the respondents who reporting always/usually receiving a vaccination, 84.6% were vaccinated in 2009–20010. 30.8% and 6.3% of respondents who reported sometimes or rarely receiving an influenza vaccination were vaccinated in 2009–2010 respectively (p<.01) (Table 2).

**Table 2:** Vaccination History of Respondents Vaccinated in 2009–2010 (Weighted)*.

	Overall (%)	Always/UsuallyN = 1587 (%)	SometimesN = 126 (%)	RarelyN = 461 (%)	First time in2009–2010 N = 136 (%)	p-value**
Current Vaccination (2009–2010)	39.1	84.6	30.8	6.3	100.0	<.01
*Location of Vaccination*						
Medical setting	47.5	46.6	80.9	57.5	51.7	.03
Retail setting	17.0	19.6	0	5.3	17.2	.01
Workplace	19.5	17.8	10.0	7.4	15.5	.42
*Timing of Vaccination*						
Aug-Sept	20.0	18.2	10.2	32.6	20.0	<.01
Oct-Nov	67.0	69.5	77.4	57.4	54.2	<.01
December-March	13.5	12.3	12.4	9.9	27.6	<.01

*The poststratification weights were computed using data from the Current Population Survey and adjust for known sampling probabilities; sample stratification; and non-response to panel recruitment and panel attrition.

**p-value for differences across always/sometimes/rarely.

Among those who were vaccinated for the first time in 2009–2010, 51.7% were vaccinated for seasonal influenza in a medical setting, 17.2% in a retail setting, and 15.5% in a workplace. Compared to those who are usually/always vaccinated, those who sometimes or rarely receive influenza vaccinations were more likely to be vaccinated in a medical setting (46.6% vs. 80.9% and 57.5%) (p = .03) in 2009–2010. The association between vaccination history and vaccination in a medical setting remained after adjusting for demographic variables including age, gender, race, and income, and membership in a group specifically recommended for seasonal influenza vaccination.

## Discussion

Our study found that a large fraction of U.S. adults have never received influenza vaccinations despite a near-universal recommendation at the time of this study and extensive outreach. In addition, the group of adults who were vaccinated for influenza in 2009–2010 displayed fairly heterogeneous behaviors with respect to past vaccine uptake and choice of vaccination locations.

National surveys report that 60% of U.S. adults remain unvaccinated in any given influenza season [12], and our results suggest that a large fraction of those are individuals who *never* get vaccinated. While encouraging this group to obtain vaccinations would have the largest impact on overall rates, the true “low hanging fruit” in a given season is the 20% that get vaccinated at least some of the time. For this group, interventions such as providing reminders and increasing access may be most cost-effective because these individuals generally hold positive views about vaccination even if they lack strong motivation to obtain annual vaccinations [6]. On the other hand, for those who have never received an influenza vaccination, we can consider interventions that incentivize first-time use, the rationale being that a one-time, positive (or even neutral) experience with vaccination will increase confidence in the safety of vaccination, heighten awareness, and ultimately create a norm of vaccination.

The associations we observed also provide preliminary insight into the role of complementary vaccination settings in supporting repeated, annual influenza vaccination. Medical settings appear important for the initiation of influenza vaccination and for reaching patients who area infrequently vaccinated. On the other hand, retail locations and workplaces disproportionately serve those who are regularly vaccinated, as individuals who always/usually receive influenza vaccinations reported greater reliance on complementary settings in 2009–2010. For those positively inclined to be vaccinated, complementary settings may serve as low-cost, convenient alternatives to traditional medical settings as well as an important sources of vaccination reminders (e.g., through retail advertising of vaccination services).

At the same time, our results also beg the question: what can complementary settings do to play a more significant role in initiating vaccination among those who never or seldom obtain vaccinations? Pharmacists, for example, could counsel all patients filling prescriptions on the importance of influenza vaccination. Healthcare provider recommendations are particularly effective at increasing immunization rates, even among disinclined or hesitant patients [5], [13], [14].

The major limitation of this study is the potential for recall bias because participants described previous vaccination behaviors. However, our results are consistent with several cross-sectional studies that have explored vaccination habits and found that history of influenza vaccination consistently predicts current vaccination across a range of populations including high risk adults [15], [16], healthcare workers [17], [18], and healthy working adults [19]. In addition, the H1N1 pandemic which began in the spring of 2009, may have impacted uptake patterns for seasonal influenza vaccine in the 2009–2010 season, thus limiting the generalizability of our results. Although this is a concern, the adult vaccination rate in the 2009–2010 season is consistent with trends since 2005 (approx. 2% increase each year) [20]. Finally, this survey was conducted prior to the universal recommendation that all individuals age 6 months and older receive annual influenza vaccinations; however, even prior to this change which occurred in 2010, 85% of the U.S. population was subject to a formal recommendation, and the remaining 15% were subject to an informal recommendation that stated, “Vaccination is recommended for any persons who wish to reduce the likelihood of their becoming ill with influenza or transmitting influenza to others should they become infected [10].”

By understanding the vaccination habits of individuals, we can more appropriately choose among interventions designed to encourage the initiation vs. maintenance of desired behaviors, and decide which types of strategies are likely to have the greatest impact on increasing vaccination uptake among adults. This study suggests that despite the growth of complementary settings for vaccination, traditional medical settings continue to play an especially critical role in vaccinating adults who do not regularly receive influenza vaccinations.
